# Insights into the interaction between hemorphins and δ-opioid receptor from molecular modeling

**DOI:** 10.3389/fmolb.2024.1514759

**Published:** 2024-12-12

**Authors:** Priya Antony, Bincy Baby, Ranjit Vijayan

**Affiliations:** ^1^ Department of Biology, College of Science, United Arab Emirates University, Al Ain, United Arab Emirates; ^2^ The Big Data Analytics Center, United Arab Emirates University, Al Ain, United Arab Emirates; ^3^ Zayed Center for Health Sciences, United Arab Emirates University, Al Ain, United Arab Emirates

**Keywords:** opioid receptors, hemorphins, molecular docking, molecular simulations, camel hemorphins

## Abstract

Hemorphins are short atypical opioid peptide fragments embedded in the β-chain of hemoglobin. They have received considerable attention recently due to their interaction with opioid receptors. The affinity of hemorphins to opioid receptors μ-opioid receptor (MOR), δ-opioid receptor (DOR), and κ-opioid receptor (KOR) has been well established. However, the underlying binding mode and molecular interactions of hemorphins in opioid receptors remain largely unknown. Here, we report the pattern of interaction of camel and other mammalian hemorphins with DOR. Extensive *in silico* docking and molecular dynamics simulations were employed to identify intermolecular interactions and binding energies were calculated to determine the affinity of these peptides for DOR. Longer forms of hemorphins - hemorphin-7, hemorphin-6, camel hemorphin-7, and camel hemorphin-6 had strong interactions with DOR. However, camel hemorphin-7 and camel hemorphin-6 had high binding affinity towards DOR. Thus, the findings of this study provide molecular insights into how hemorphins, particularly camel hemorphin variants, could be a therapeutic agent for pain regulation, stress management, and analgesia.

## 1 Introduction

Hemorphins are a group of endogenous opioid peptides derived from the β-chain of hemoglobin (Gomes et al., 2010; Ali et al., 2019; Ali et al., 2020). These bioactive peptides have received considerable attention due to their potential therapeutic effects on spatial learning, analgesia, inflammation and antihypertension (Davis et al., 1989; Sanderson et al., 1998; Dale et al., 2005; Cheng et al., 2012; Ayoub and Vijayan, 2021; Hung et al., 2021). Opioid receptors are G protein-coupled receptors (GPCR) characterized by the presence of the archetypical seven transmembrane helices. The μ-opioid receptor (MOR) is the primary target of opioid analgesics while δ-opioid receptor (DOR) and κ-opioid receptor (KOR) are also involved in the regulation of pain and analgesia (Waldhoer et al., 2004; Valentino and Volkow, 2018). The affinity of hemorphins to opioid receptors plays a crucial role in regulating pain perception (Mielczarek et al., 2021).

Hemorphins peptides vary in size from 4 to 10 amino acids (Ianzer et al., 2006). Hemorphin peptides share a core tetrapeptide sequence of tyrosine-proline-tryptophan-threonine (YPWT). Hemorphin-4 (hem-4) was the first hemorphin characterized from bovine blood. Variations of N-terminal and C-terminal extensions of the core sequence have been isolated from human and bovine tissues (Ayoub and Vijayan, 2021). Various forms of hemorphin peptides including, hemorphin-4, hemorphin-6, hemorphin-7, LVV-hemorphin-6, LVV-hemorphin-7, and VV-hemorphin-7 showed partial to full binding in a competitive manner with the endogenous opioid-related peptides such as enkephalins and dynorphins in radioligand experiments. Different forms of hemorphins including synthetic and purified hemorphin peptides from human and bovine blood and brain tissues show varying affinities for opioid receptors (Liebmann et al., 1989; Zadina et al., 1990; Garreau et al., 1995; Zhao et al., 1997; Szikra et al., 2001).

In humans, MOR, DOR, and KOR share around 60% sequence identity indicating a conserved structure supporting its functional roles. Hence, a hypothesis worth testing is whether hemorphins could bind to DOR and KOR in a manner similar to MOR. The interaction of hemorphins with μ-opioid receptor (MOR) has been well studied (Davis et al., 1989; Garreau et al., 1998; Ali et al., 2020). Hemorphin-4 and hemorphin-5 have been shown to inhibit nociception by acting on MOR (Davis et al., 1989). Furthermore, VV-hemorphin-7 and LVV-hemorphin-7 were also shown to bind with the same potency as hemorphin-4 and hemorphin-5 but with less potency than hemorphin-6 and hemorphin-7 (Garreau et al., 1995). We previously reported the interaction of LVV-hemorphin-7 with MOR (Ali et al., 2019). Identification of a similar mode of interaction would suggest that hemorphins could assist with antinociceptive, antidepressant and sedative effects via its action on DOR, which could then be investigated further. The δ-opioid receptor mediates its effects through Gi/o protein signaling, reducing cAMP levels, inhibiting calcium channels, and activating potassium channels, resulting in neuronal inhibition and analgesia (Valentino and Volkow, 2018; Mielczarek et al., 2021). Additional pathways, including PLC activation and β-arrestin signaling, further contribute to its antinociceptive, antidepressant, and sedative effects by modulating neurotransmitter release and synaptic plasticity (Waldhoer et al., 2004). As potential DOR agonists, hemorphins can utilize these pathways to produce therapeutic effects, offering a promising route for the development of safer and more effective analgesics and antidepressants.

**FIGURE 1:**
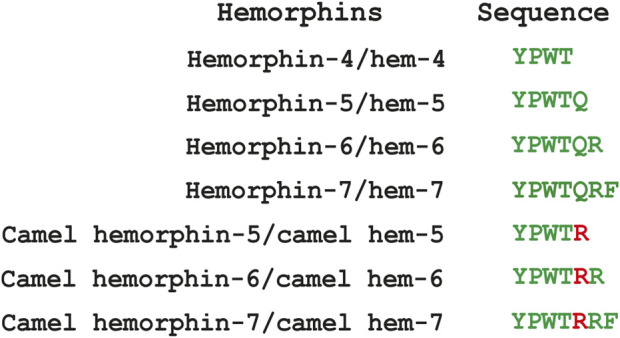
List of hemorphins docked with δ-opioid receptor.

The hemorphin sequence is well-conserved among mammals, except camels, which uniquely feature a Q > R variation following the shared YPWT sequence. We also studied and reported this single amino acid disparity in camels on several targets using *in silico* and *in vitro* techniques. The camel forms of these peptides were reported to exhibit a greater affinity for all the protein targets tested (Ali et al., 2019; Ali et al., 2020; Jobe et al., 2023). The primary objective of this study was to identify and understand the molecular mechanisms underlying the interaction of hemorphins of camels and that of other mammals with DOR using *in silico* docking and molecular dynamics simulations. Figure 1 lists the sequence of hemorphins used in this study.

## 2 Materials and methods

### 2.1 Protein structure preparation

The crystal structure of DOR (PDB ID: 6PT2) was obtained from the Protein Data Bank (PDB). The structure was prepared for *in silico* docking using the protein preparation wizard of Schrödinger suite 2022–2 (Wu et al., 2012; Claff et al., 2019; Schrodinger, 2024). The protein structure was preprocessed by adding hydrogens, removing unwanted water molecules, assigning proper bond orders, adjusting the ionization state, disoriented group orientation, disulfide bond addition, partial charge assignment, and fixing residues with missing atoms and side chains. The unwanted ligands and chains were removed while the tautomeric states were generated at pH 7.0. Finally, the structure of the proteins were further optimized and minimized by OPLS_2005 force field in order to preserve geometric structural stability (Madhavi Sastry et al., 2013).

### 2.2 Binding site identification and grid generation

A receptor grid was generated for site-specific docking by identifying the location of the co-crystallized ligand in the protein structure. OPLS 2001 force field was used to represent the protein. Default parameters were used and the van der Waals radii of the receptor atom were scaled to 1.0 and the partial charge cutoff value was set to 0.25.

### 2.3 Peptide docking

Peptide docking was performed to determine the most likely binding orientation of hemorphins within DOR, and to analyze the resulting intermolecular interactions and the binding free energy. Standard precision flexible docking was performed for docking the peptide using Schrödinger Glide version 2022–2 with default parameters (Friesner et al., 2004). The docked poses were ranked in using the GlideScore (GScore) scoring function (Friesner et al., 2006). The poses with lowest GScore value were selected for further analysis.

### 2.4 Binding free energy calculation

After docking, the best docked poses were taken to analyze the various types of contacts including hydrogen bonds, hydrophobic interactions, salt bridges, π–π stacking and π-cation interactions. The best-docked poses were subjected to molecular mechanics generalized Born surface area (MM-GBSA) calculations to evaluate the binding free energy in an implicit solvent model. MM-GBSA binding energy was calculated using Schrödinger Prime employing the OPLS 2005 force field and the VSGB 2.0 implicit solvent model (Li et al., 2011; Prime, 2022).

### 2.5 Molecular dynamics (MD) simulations

MD simulations of the best docked pose of the top ranked non-camel and camel hemorphins were performed to evaluate the stability and dynamics of the binding conformation of the peptides. MD simulations were carried out using the Desmond simulation package of Schrödinger LLC with the OPLS 2005 force field (Desmond, 2022). 250 nanoseconds (ns) MD simulations were performed in triplicate for the best docked pose of camel and non-camel hemorphins with DOR. The three runs of each of the peptide bound complexes were performed with different initial velocities. The complexes of receptors with the best docked non-camel and camel hemorphins were embedded into a pre-equilibrated DPPC membrane in an orthorhombic box (Matyszewska and Bilewicz, 2008). All systems were solvated with a water box, using SPC water model with a buffer distance of 10 Å (Mark and Nilsson, 2001). The simulation models were neutralized with the required number of counterions, and the salt concentration was set at 0.15 M NaCl.

The systems were subjected to steepest descent minimization with Desmond’s default protocol prior to performing the MD simulations. All systems were first relaxed using the default relaxation protocol for membrane proteins which consists of eight stages (Zhang, 2015; Ali et al., 2019). After relaxation, 250 ns simulation run was performed for each system using the default simulation protocol. The simulations were performed under NPT ensemble and the systems were maintained at a constant temperature of 300 K using the Nose-Hoover thermostat. Isotropic Martyna–Tobias–Klein barostat was used to maintain a pressure of 1 atm (Martyna et al., 1992; Martyna et al., 1994; Ikeguchi, 2004). Long and short range Coulombic interactions were analyzed with a cut-off distance of 9.0 Å using the smooth particle mesh Ewald (PME) method and short-range method (Essmann et al., 1995). A time-reversible reference system propagator algorithm (RESPA) integrator was used with an inner time step of 2.0 fs and an outer time step of 6.0 fs(Humphreys et al., 1994). RMSD, RMSF, and protein-ligand contacts of the complexes were evaluated from the trajectories and the plots were generated using R version 3.6.3.

## 3 Results

### 3.1 Molecular docking

Molecular docking was employed to identify energetically favorable binding modes of hemorphins in DOR. All possible binding modes of non-camel and camel hemorphins with DOR were predicted using peptide docking method of Schrödinger suite of tools. The GlideScore scoring function was used to identify the best docked pose. Binding free energy (ΔG_bind_) for the binding of peptides with the protein were estimated using Prime molecular mechanics with generalized Born and surface area (MM-GBSA) method. Table 1 shows the binding scores and the residues of DOR that interacted with hemorphins.

**TABLE 1:** Molecular docking results of hemorphins with δ-opioid receptor. A represents DOR and B represents hemorphin.

Hemorphin	GlideScore (kcal/mol)	MM-GBSA binding energy (kcal/mol)	Hydrogen bonds	π-π interactions	π-cation interactions	Salt bridges	Hydrophobic interactions
Hemorphin-7	−11.64	−82.89	B:Tyr1 -A:Gln105 B:Tyr1-A:Asp128 B:Gln5-A:Ser206 B:Gln5-A:Asp210 B:Arg6-A:Asp108 B:Phe7-A:Arg291	B:Trp3-A:Trp284	B:Phe7-A:Arg291	B:Tyr1-A:Asp128 B:Arg6-A:Asp108	B:Tyr1-A:Ile277 B:Pro2-A:Gln105 B:Trp3-A:Phe280 B:Trp3-A:Trp284 B:Trp3-A:Leu300 B:Thr4-A:Leu125 B:Thr4-A:Leu200 B:Phe7-A:Trp284
Hemorphin-6	−11.18	−77.82	B:Tyr1-A:Asp128 B:Thr4-A:Cys198 B:Gln5-A:Arg291 B:Arg6-A:Asp210 B:Arg6-A:Arg192	B:Trp3-A:Trp284		B:Tyr1-A:Asp128	B:Tyr1-A:Met132 B:Tyr1-A:Val217 B:Tyr1-A:Val281 B:Pro2-A:Ile304 B:Trp3-A:Phe280 B:Trp3-A:Val281 B:Trp3-A:Trp284 B:Trp3-A:Leu300 B:Thr4-A:Phe202
Hemorphin-5	−10.05	−73.1	B:Tyr1-A:Asp128 B:Tyr1-A:Ile304 B:Thr4-A:Cys198 B:Gln5-A:Asp108 B:Gln5-A:Arg291	B:Tyr1-A:Trp274 B:Trp3-A:Trp284		B:Tyr1-A:Asp128	B:Tyr1-A:Met132 B:Tyr1-A:Trp274 B:Tyr1-A:Ile277 B:Tyr1-A:Tyr308 B:Pro2-A:Lys214 B:Trp3-A:Phe280 B:Trp3-A:Trp284 B:Trp3-A:Leu300
Hemorphin-4	−8.54	−73.32	B:Tyr1-A:Asp128 B:Thr4-A:Cys198 B:Thr4-A:Arg291	B:Trp3-A:Trp284		B:Tyr1-A:Asp128 B:Thr4-A:Arg291	B:Tyr1-A:Met132 B:Tyr1-A:Trp274 B:Tyr1-A:Tyr308 B:Pro2-A:Lys214 B:Trp3-A:Phe280 B:Trp3-A:Trp284 B:Trp3-A:Leu300
Camel hemorphin-7	−10.78	−91.93	B:Tyr1-A:Asp128 B:Arg5-A:Asp108 B:Arg5-A:Glu112 B:Phe7-A:Arg291	B:Tyr1-A:Trp274 B:Tyr1-A:Tyr308 B:Trp3-A:Trp284	B:Tyr1-A:Tyr129	B:Tyr1-A:Asp128 B:Arg5-A:Asp108	B:Tyr1-A:Met132 B:Tyr1-A:Trp274 B:Tyr1-A:Ile304 B:Tyr1-A:Tyr308 B:Pro2-A:Val217 B:Pro2-A:Val281 B:Trp3-A:Phe280 B:Trp3-A:Val281 B:Trp3-A:Trp284 B:Trp3-A:Leu300 B:Phe7-A:Trp284
Camel hemorphin-6	−10.39	−80.34	B:Tyr-A:Asp128 B:Arg5-A:Asp108 B:Arg5-A:Glu112	B:Tyr1-A:Ile304 B:Trp3-A:Trp284		B:Tyr1-A:Asp128 B:Tyr1-A:Tyr129 B:Arg6-A:Asp210	B:Tyr1-A:Trp274 B:Tyr1-A:Ile277 B:Tyr1-A:Tyr308 B:Pro2-A:Lys214 B:Pro2-A:Val217 B:Pro2-A:Val281 B:Trp3-A:Val281 B:Trp3-A:Trp284 B:Trp3-A:Phe280 B:Trp3-A:Leu300
Camel hemorphin-5	−9.36	−60.57	B:Tyr1-A:Asp128 B:Thr4-A:Leu200 B:Arg5-A:Asp108	B:Tyr1-A:Trp274 B:Tyr1-A:Tyr308 B:Trp3-A:Trp284		B:Tyr1-A:Asp128 B:Arg5-A:Asp108	B:Tyr1-A:Met132 B:Tyr1-A:Trp274 B:Tyr1-A:Ile304 B:Tyr1-A:Tyr308 B:Pro2-A:Ile304 B:Trp3-A:Phe280 B:Trp3-A:Val281 B:Trp3-A:Trp284 B:Trp3-A:Leu300

“A” represents protein and “B” represents the peptides.

The peptides docked with a similar binding pose within DOR. The longer forms of hemorphin, hemorphin-7 (hem-7), camel hemorphin-7 (camel hem-7), hemorphin-6 (hem-6), and camel hemorphin-6 (camel hem-6) showed strong interactions with DOR. Interestingly, the camel variants, camel hem-7 and camel hem-6 showed higher binding affinity towards DOR based on binding free energy calculation (Table 1). The interactions observed in the docking provide insights into their binding mechanisms.

**FIGURE 2:**
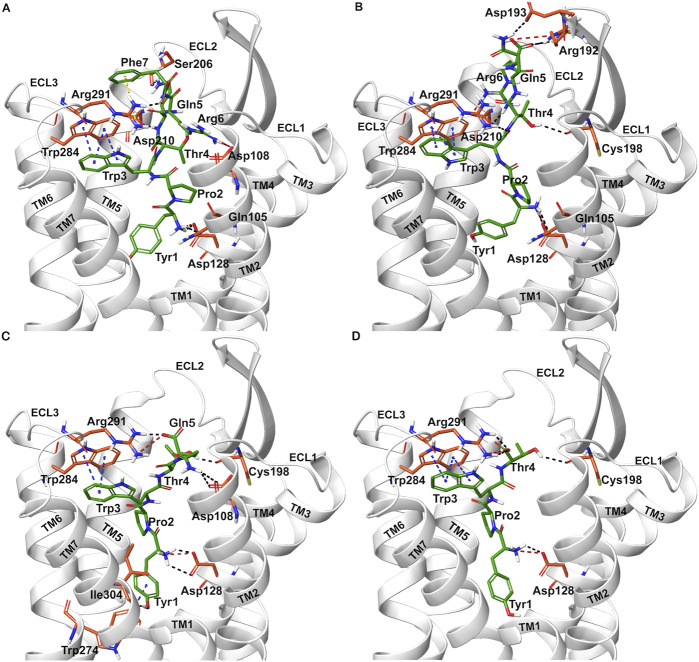
Interaction of hemorphin variants with δ-opioid receptor (DOR). **(A)** DOR/hem-7 **(B)** DOR/hem-6 **(C)** DOR/hem-5 **(D)** DOR/hem-4.

**FIGURE 3:**
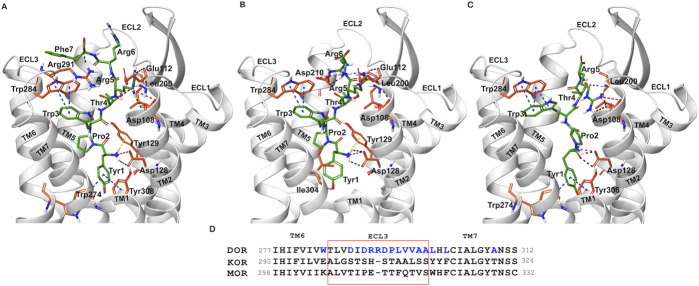
Interaction of camel hemorphin variants with δ-opioid receptor (DOR). **(A)** DOR/camel hem-7 **(B)** DOR/camel hem-6 **(C)** DOR/camel hem-5. **(D)** Opioid receptor sequence alignment of the nonconserved ECL3 (light red box) and the region close to the extracellular ends of TM5 and TM7. The nonconserved residues are shown in blue color.

Docked poses of hemorphins with DOR revealed that the N terminal of hemorphins bound deeply within the binding pocket and forms critical interactions which are essential for the activation of the receptor. The binding pocket is located deep within the transmembrane helices that includes transmembrane helix 3 (TM3), transmembrane helix 5 (TM5), transmembrane helix 6 (TM6) and transmembrane helix 7 (TM7). The N-terminal tyrosine made contributed to the binding affinity for DOR due to its ability to form key interactions within the receptor binding pocket. Docked poses of all the hemorphins with DOR revealed that Tyr1 was embedded deep in the binding pocket, forming hydrogen bond with Asp128. Importantly, the protonated amino group of Tyr1 formed salt bridge interaction with Asp128 in all the docked poses of hemorphins with DOR (Figure 2). Asp128 is conserved among all the opioid receptors and is a key residue in the activation of DOR (Supplementary Figure S1). Interestingly, Tyr1 formed π-π interaction and hydrophobic interaction with Tyr308 in the complex structure of DOR with camel hemorphin variants (Figure 3; Supplementary Figures S2B, S2D, S3B).

Interactions with transmembrane helices, particularly the extracellular ends of transmembrane helices 6 (TM6), transmembrane helices 7 (TM7) and the extracellular loop 3 (ECL3) play a pivotal role in the selectivity of peptides within the binding pocket. The hemorphins, hem-7 and camel hem-7 exhibited similar pattern of interaction with DOR. In both cases, Phe7 of both hem-7 and camel hem-7 engaged with several key interactions that significantly contributed to the binding affinity of the peptides. In both cases, Phe7 formed a hydrogen bond with the side chain of Arg291 in ECL3 (Figures 2A, 3A). In hem-7, the phenyl ring of Phe7 forms a π-cation interaction with the side chain of Arg291 (Figure 2A). Additionally, the aromatic ring of Phe7 in both hem-7 and camel hem-7 makes hydrophobic contact with the nonconserved Trp284 in the extracellular ends of TM6 (Supplementary Figures S2A, S2B). These interactions involving the phenyl ring enhance the binding strength. Additionally, Trp3 formed multiple contacts within a hydrophobic pocket formed by Ile277 (TM6), Phe280 (TM6), Val281 (TM6), Trp284 (TM6), Ile289 (ECL3), Arg291 (ECL3), and Leu300 (TM7). Specifically, in hem-7, Trp3 interacted with Phe280, Trp284, and Leu300, whereas in camel hem-7, Trp3 makes hydrophobic contacts with Phe280, Val281, Trp284, and Leu300 (Table 2 and Supplementary Figures 2A, 2E). Additionally, π-π interaction was observed between Trp3 and the nonconserved Trp284 (TM6) in both DOR/hem-7 and DOR/camel hem-7 complexes (Figures 2A, 3A). Figure 3D shows the conserved and nonconserved residues of ECL3 and the extracellular ends of helices 6 and 7 (Figure 3D). The π-π interaction is significant as it provides additional stabilization through stacking interactions of aromatic rings, contributing to the overall binding affinity of the peptides. In the hem-6 bound structure of DOR, Gln5 formed a hydrogen bond with Arg291 (ECL3) (Figure 2B). Similar to hem-7, Trp3 in hem-6 exhibited extensive hydrophobic interaction with Phe280 (TM6), Val281 (TM6), Trp284 (TM6), and Leu300 (TM7) and also formed a π-π interaction with Trp284 (TM6) (Figure 2B; Supplementary Figure S2C). In the docked pose of DOR/camel hem6, Trp3 formed hydrophobic contacts with Phe280, Val281, Trp284, and Leu300, paralleling the interactions observed in hem-6 with DOR (Table 1; Supplementary Figure S2D). This clustering of hydrophobic residues around Trp3 enhances the binding strength of the peptides. The π-π interaction between Trp3 and Trp284 in both DOR/hem-6 and DOR/camel hem-6 underscores the importance of this interaction in the binding mechanism (Figures 2B, F). In the case of the interaction of hem-5 with DOR, Gln5 formed hydrogen bond with Arg291 (Figure 2C). Similar to the abovementioned peptides, Trp3 of hem-5 and camel hem-5 engaged in a π-π interaction with Trp284 and showed hydrophobic contacts with Phe280, Val281, Trp284, and Leu300 (Figure2C, G; Supplementary Figures S3A, S3B). In the case of hem-4, Thr4 formed both a hydrogen bond and a π-cation interaction with Arg291. Additionally, Trp3 in hem-4, much like in hem-5, engaged in a π-π interaction with Trp284 and formed hydrophobic contacts with Phe280, Trp284, and Leu300 (Figure 2D; Supplementary Figure S3C). These interactions further emphasize the significance of Trp3 across different hemorphins, highlighting its central role in mediating binding through both π-π interactions and hydrophobic contacts.

The N-terminal region (Tyr1-Pro2-Trp3) of hemorphins exhibited hydrophobic interactions with either TM5 or TM7, or both, penetrating deeply into the binding pocket of DOR. The key residues involved in these interactions are Lys214 and Val217 in TM5, and Leu300, Ile304, and Tyr308 in TM7. In the docked pose of hem-7 with DOR, Trp3 formed hydrophobic interactions with Leu300 (TM7) (Supplementary Figure S2A). In the DOR/hem-6 docked structure, Tyr1, Pro2 and Trp3 showed hydrophobic interactions with Val217 (TM5), Ile304 (TM7), and Leu300 (TM7) respectively (supplementary Figure S2C). In the case of hem-5, Tyr1 interacted with Tyr308, Pro2 interacted with Lys214 (TM5), and Trp3 with Leu300 (TM7) (Supplementary Figure S3A). Similarly, in the docked pose of DOR with hem-4, Pro2 interacted hydrophobically with Lys214, while Tyr1 and Trp3 engaged with Tyr308 (TM7) and Leu300 (TM7) respectively (Supplementary Figure S3C). Hemorphin variants from camel displayed more extensive hydrophobic contacts with both TM5 and TM7. In DOR/camel hem-7, Tyr1 interacted with Ile304 (TM7) and Tyr308 (TM7), Pro2 with Val217 (TM5), and Trp3 with Leu300 (TM7) while in the case of DOR/camel hem-6, Tyr1 showed hydrophobic contacts with Tyr308, Pro2 engaged with Lys214 and Val217 (TM5), and Trp3 with Leu300 (TM7) (Supplementary Figures S2B, S2C). The hydrophobic interactions of camel hem-5 towards TM5 and TM7 include Tyr1 with Ile304 (TM7), Pro2 with Ile304 (TM7), and Trp3 with Leu300 (TM7) (Supplementary Figure S3B).

### 3.2 Molecular dynamics simulation

**FIGURE 4:**
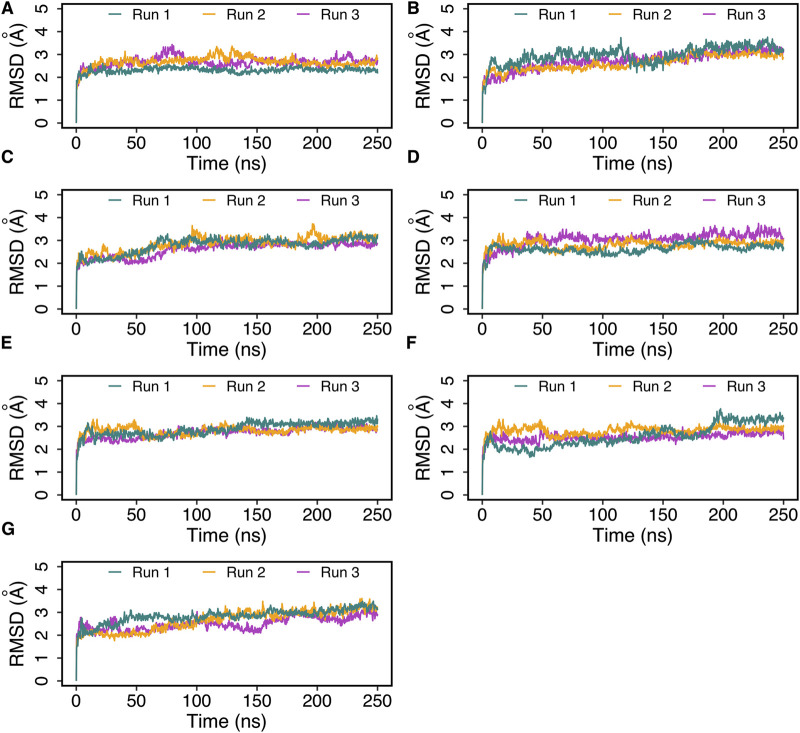
Root mean square deviation (RMSD) of DOR complexed with hemorphins obtained from triplicate 250 ns MD simulations. **(A)** DOR/hem-7, **(B)** DOR/camel hem-7, **(C)** DOR/hem-6, **(D)** DOR/camel hem-6, **(E)** DOR/hem-5, **(F)** DOR/camel hem-5, and **(G)** DOR/hem-4.

**FIGURE 5:**
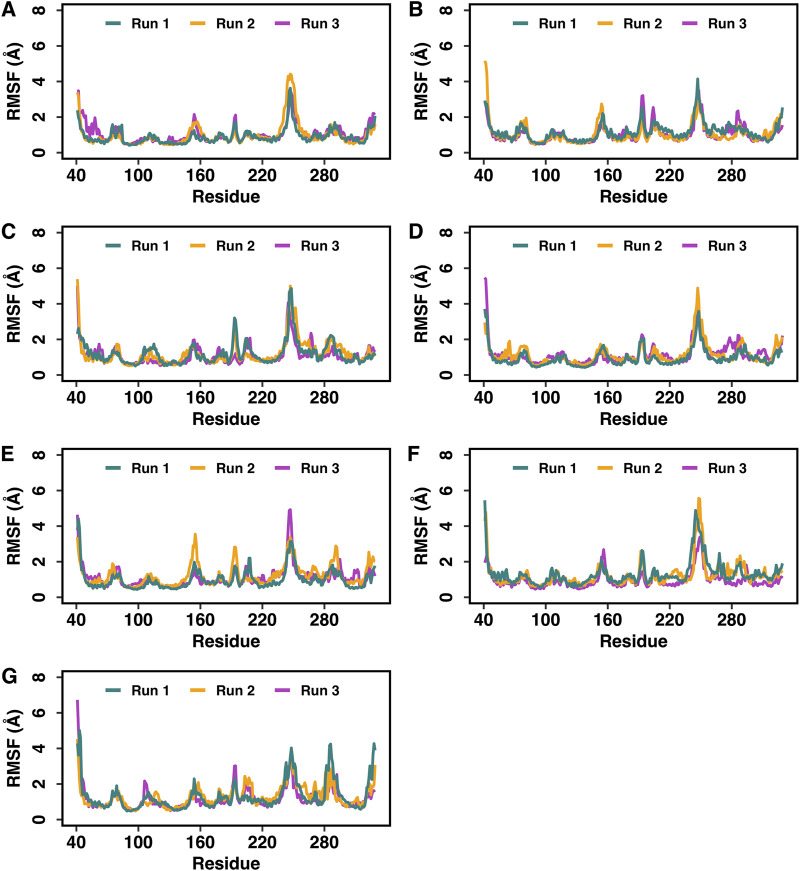
Root mean square fluctuations (RMSF) of the Cα atoms of DOR in the DOR-hemorphin complexes obtained from triplicate 250 ns MD simulations. **(A)** DOR/hem-7, **(B)** DOR/camel hem-7, **(C)** DOR/hem-6, **(D)** DOR/camel hem-6, **(E)** DOR/hem-5, **(F)** DOR/camel hem-5, and **(G)** DOR/hem-4.

The best docked poses of hemorphin variants within the DOR binding site were subsequently subjected to molecular dynamics (MD) simulations to study the structural dynamics of the complexes. MD simulations were performed for 250 ns in triplicates to evaluate the stability of the complex and the bound peptides. The simulation trajectories refined the peptide binding pose, ultimately leading to a more accurate representation of the dynamics of the peptide-protein interactions. The root mean square deviation (RMSD) of protein C-alpha (Cα) atoms was evaluated to study the difference in protein conformation in each frame of the MD trajectory when compared to the initial structure. An assessment of the RMSD provides insights into the flexibility and conformational changes that happened to protein upon ligand binding. Figure 4 shows the change in protein RMSD in the presence of hemorphins in MD simulations for 250 ns? In all the complexes, the system stabilized under 4 Å after a few nanoseconds (Figure 4). The peptides reached a relatively stable conformation after an initial period of adjustment during the simulation. These results suggest that the binding of hemorphins with human δ-opioid receptor was stable. To investigate the residue-level protein flexibility for each system, the root mean square fluctuation (RMSF) values of backbone atoms were evaluated. Figure 5 illustrates the fluctuations of each residue in the protein structure. Based on the calculated RMSF values as depicted in Figure 5, it was noted that the intracellular loop 3 (ICL3) region, spanning from Arg239 to Arg261, which connects TM5 and TM6, exhibited the largest fluctuations. This regions is not a part of the binding region of DOR. Furthermore, the flexibility of the extracellular loop 3 (ECL3) region (Thr285 to Ala299) in DOR/hem4 complex is greater compared to other hemorphins complexed with DOR (Figure 5G).

Specific interactions between the hemorphin variants and DOR were analyzed based on their persistence for over 50% of the total simulation time. The persistence indicates that these interactions are likely crucial for the stability and function of the complex.

**FIGURE 6:**
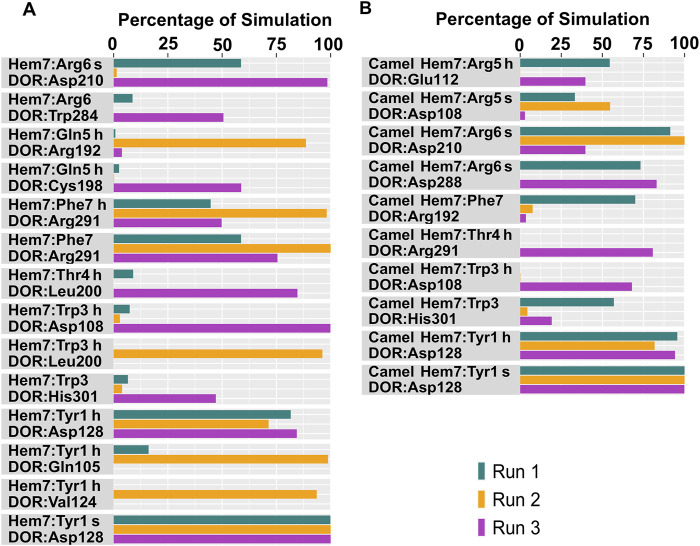
Polar interactions between hemorphin variants and δ-opioid receptor. **(A)** DOR/hem-7 and **(B)** DOR/camel hem-7. “H” denotes a hydrogen bond and “s” denotes a salt bridge.

**FIGURE 7:**
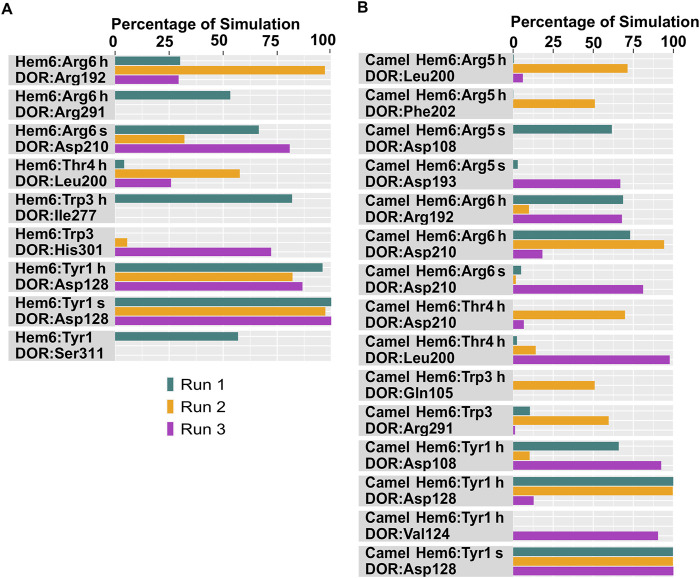
Polar interactions between hemorphin variants and δ-opioid receptor. **(A)** DOR/hem-6, and **(B)** DOR/camel hem-6. “H” denotes a hydrogen bond and “s” denotes a salt bridge.

**FIGURE 8:**
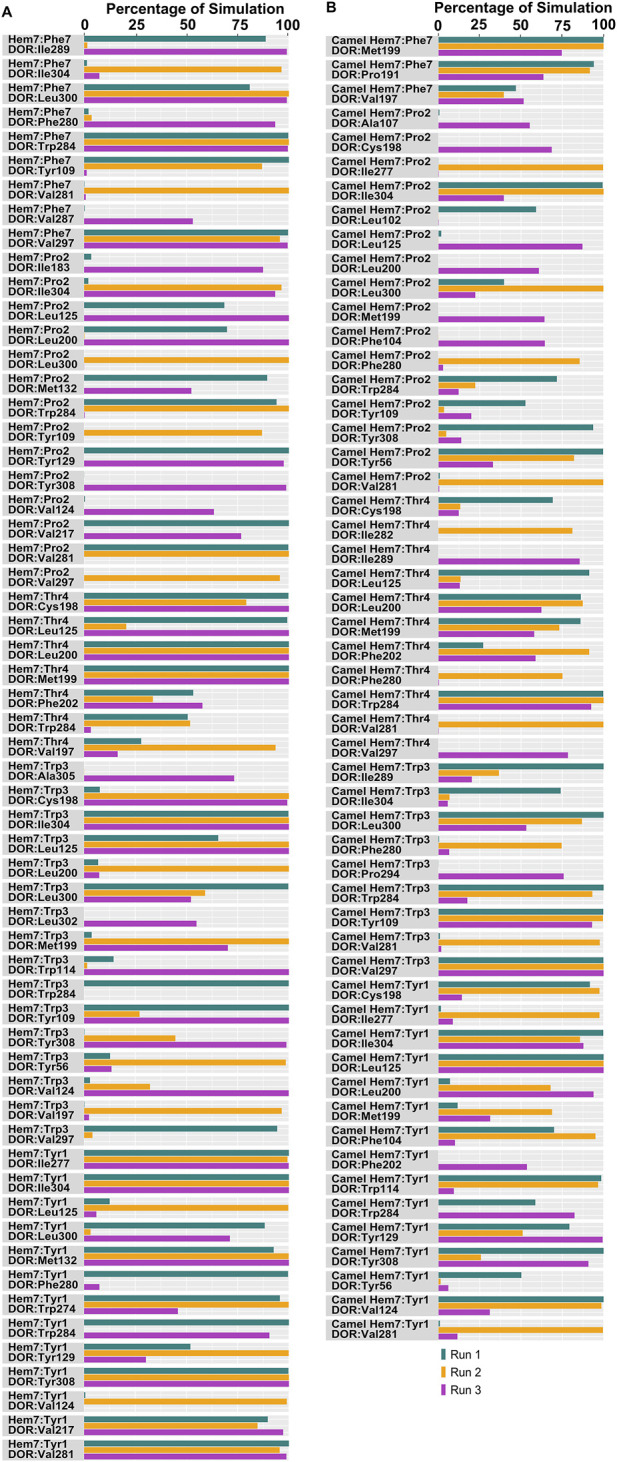
Hydrophobic interactions observed between hemorphin variants and δ-opioid receptor. **(A)** DOR/hem-7 and **(B)** DOR/camel hem-7.

**FIGURE 9:**
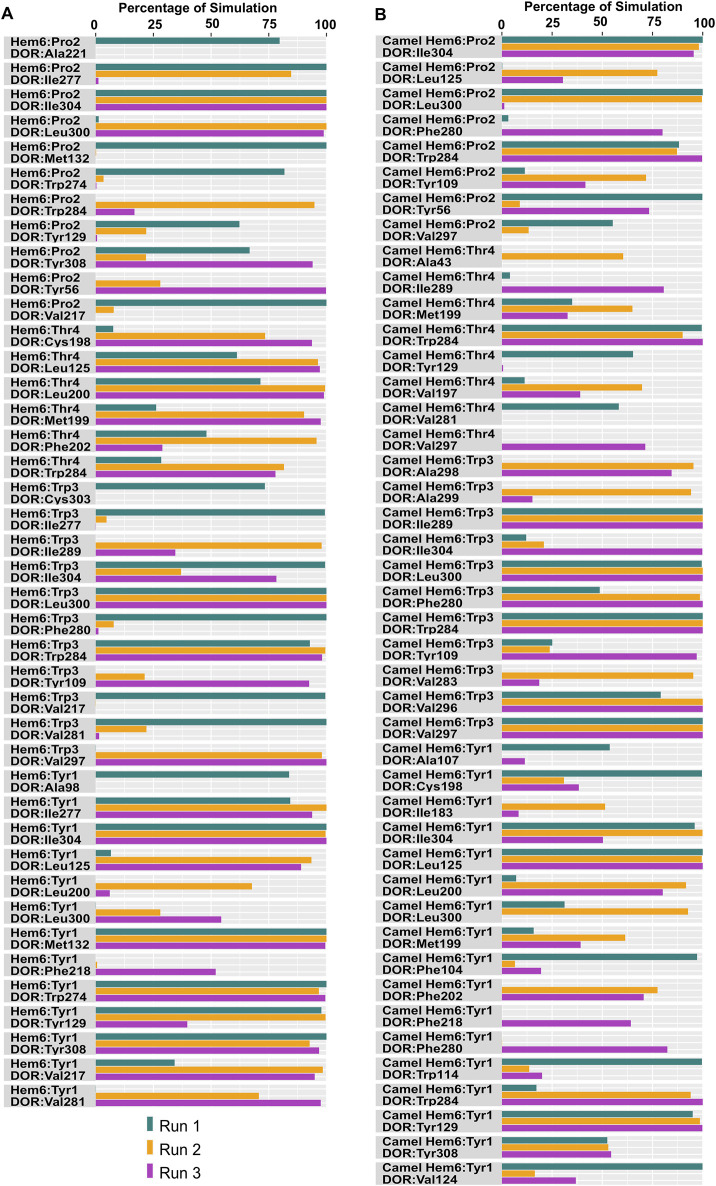
Hydrophobic interactions observed between hemorphin variants and δ-opioid receptor. **(A)** DOR/hem-6 and **(B)** DOR/camel hem-6.

Tyr1 of all the hemorphin variants bound deeper into the binding pocket of DOR and the protonated amino group of Tyr1 in each hemorphin consistently interacted with the conserved Asp128 throughout the simulation (Figures 6, 7; Supplementary Figure S4, S5). The interactions of Tyr1, particularly, the salt bridge interaction with Asp128 were maintained throughout the simulation in all the runs, indicating a strong electrostatic interaction between the hemorphin variants and DOR. Furthermore, hydrophobic interactions of Tyr1 particularly with Tyr129 and Tyr308 were consistently maintained throughout the simulation period of all hemorphin complexes except DOR/camel hem-5, further enhancing the stability of Tyr1 in the binding pocket (Figures 8 and 9; Supplementary Figure S4, S5). These stable interactions suggest that Tyr1 plays a critical role in anchoring the hemorphins to DOR, contributing to the strong binding affinity of the complex.

In the DOR/hem-7complex, the interaction between Phe7 and the nonconserved Arg291 in ECL3 was found to be stable, involving both π-cation and hydrogen bond (Figure 6A). The persistence of these interactions during the simulation suggests that the interaction of Phe7 with the nonconserved Arg291 is a key contributor to the stability of the complex. Hydrophobic interactions of Phe7 with Val281 (TM6), Trp284 (TM6), Val287 (ECL3), Ile289 (ECL3), Val297 (ECL3), and Leu300 (TM7) were consistently maintained in the simulations. Similarly, Trp3 maintained hydrophobic interactions with the nonconserved Trp284 (TM6) and Leu300 (TM7), further enhancing the stability of the complex (Figure 8A). In MD simulations of DOR with camel hem-7, Phe7 formed a hydrogen bond with Arg192 (ECL2), Thr4 established a hydrogen bond with Arg291 in ECL3 and a salt bridge was observed between Arg6 and Asp288 (ECL3) (Figure 6B). Phe7 showed consistent hydrophobic interactions with Pro191, Val197, and Met199 in ECL2 whereas Trp3 maintained multiple hydrophobic contacts with Val281 (TM6), Trp284 (TM6), Ile289 (ECL3), Val297 (ECL3), and Leu300 (TM7) and Thr4 also involved in hydrophobic interactions with Val281 (TM6), Trp284 (TM6), Ile289 (ECL3), and Val297 (ECL3) (Figure 8B). Arg6 of hem6 formed a hydrogen bond with Arg291, while Thr4 and Pro2 formed hydrophobic contacts with Trp284 (TM6) (Figure 7A). Trp3 consistently engaged in hydrophobic interactions with Ile277 (TM6), Val281 (TM6), Trp284 (TM6), Ile289 (ECL3), Val297 (ECL3), and Leu300 (TM7), underscoring its significant role in stabilizing the DOR/hem-6 complex (Figure 9A). In the DOR/camel hem-6 complex, Trp3 formed a π-cation interaction with Arg291 in ECL3 and also formed hydrophobic interactions with Trp284 (TM6), and Leu300 (TM7) as well as residues in ECL3, including Ile289, Val296, Val297, Ala298, and Ala299, while Thr4 displayed hydrophobic interactions with Val281 (TM6), Trp284 (TM6), Ile289 (ECL3), and Val297 (ECL3) (Figures 7B, 9B). In the DOR/hem-5 complex, Gln5 formed a hydrogen bond with Arg291 (ECL3) and Arg292 (ECL3) and Trp3 exhibited a π-π interaction with His301 (Supplementary Figure 4A). Trp3 consistently maintained hydrophobic interactions with multiple residues, including Ile277 (TM6), Val281 (TM6), Trp284 (TM6), Ile289 (ECL3), Val296 (ECL3), Val297 (ECL3), and Leu300 (TM7), emphasizing its significant role in stabilizing the DOR/hem-5 complex. Additionally, Thr4 formed hydrophobic contacts with Trp284 (TM6) and Ile289 (ECL3), while Pro2 maintained hydrophobic interactions with Trp284 (TM6) and Ile277 (TM6) further contributing to the stability of the complex (Supplementary Figure 5A). In the DOR/camel hem-5 complex, Trp3 maintained hydrophobic interactions with Ile277 (TM6), Val281 (TM6), Trp284 (TM6), Ile289 (ECL3), Val296 (ECL3), Val297 (ECL3) and Leu300 (TM7). Thr4 exhibited hydrophobic interactions with Ile289 in ECL3 and Trp284 in TM6, while Trp3 also engaged in a π-π interaction with Trp284 (Supplementary Figure 4B, 5B). Thr4 formed a hydrogen bond with Arg291 and Trp3 exhibited a π-π interaction with His301 (TM7) throughout the simulation in the DOR/hem-4 complex (Supplementary Figure 4C). Trp3 and Thr4 maintained the hydrophobic interactions with Ile277 (TM6), Val281 (TM6), Trp284 (TM6), and Leu300 (TM7) (Supplementary Figure 5C). Hydrophobic interactions with the nonconserved ECL3 region and the nonconserved residues at the extracellular ends of TM6 and TM7 were prominent in the simulation. Trp3 was observed to maintain multiple hydrophobic contacts with ECL3, TM6, and TM7. Notably, interactions with the nonconserved residues, Trp284 (TM6), Arg291 (ECL3), Val297 (ECL3), Ala298 (ECL3) and Leu300 (TM7) were consistently maintained throughout the simulation. This consistency suggests that these interactions likely play a crucial role in contributing to the overall stability of the hemorphins within DOR. RMSF calculations revealed that the ECL3 region (285–299) in the DOR/hem4 complex exhibited greater flexibility compared to other hemorphin variants (Figure 5G). Except hem4, all the other hemorphin variants showed more stable interactions with ECL3, resulting in reduced fluctuations of ECL3 in their respective DOR complexes during the simulation (Figure 5).

MD simulations confirmed the stability of the hydrophobic interactions of the N-terminus of hemorphins with TM5 and TM7. Tyr1-Pro2-Trp3 of hem-7 formed hydrophobic interactions with Leu300, Ile304, and Tyr308 in TM7, while Tyr1 and Pro2 interacted with Val217 in TM5. Additionally, Phe7 also produced hydrophobic interactions with Leu300 and Ile304 in TM7 (Figure 8A). In the DOR/hem-6 complex, Tyr1-Pro2 interacted with Tyr308 (TM7), while Tyr1-Pro2-Trp3 interacted with Val217 (TM5), Leu300 (TM7), and Ile304 (TM7) throughout the simulation. Specifically, the interactions between Tyr1 and Tyr308 (TM7), Trp3 and Leu300 (TM7), as well as Tyr1 and Pro2 with Ile304 (TM7) persisted throughout the course of the simulations (Figure 9A). In the DOR/hem-5 complex, Tyr1 and Pro2 maintained hydrophobic interactions with Val217 in TM5. Hydrophobic interactions involving Tyr1 with Ile304 (TM7) and Tyr308 (TM7), and Trp3 with Leu300 (TM7) and Ile304 (TM7) were consistently observed throughout the simulations (Supplementary Figure 5A). The simulation of DOR/hem-4 complex indicated that Tyr1-Pro2-Trp3 interacted hydrophobically with Val217 in TM5, while Tyr1 formed hydrophobic interactions with Ile304 and Tyr308 (TM7). Pro2 and Trp3 were noted to form hydrophobic interactions with Leu300 (TM7) and Ile304 (TM7) (Supplementary Figure 5C). Human hemorphin variants showed hydrophobic interactions with TM5 as well as TM7, whereas camel hemorphin variants maintained hydrophobic interactions primarily with TM7. In the DOR/camel hem-7 complex, Tyr1 maintained hydrophobic interactions with Tyr308 (TM7) and Ile304 (TM7), Pro2 interacted with Leu300 (TM7) and Tyr308 (TM7), and Trp3 showed hydrophobic interactions with Leu300 (TM7) and Ile304 (TM7) (Figure 8B). In the DOR/camel hem-6 complex, Tyr1 exhibited hydrophobic interactions with Leu300 (TM7), Ile304 (TM7), and Tyr308 (TM7), while Pro2 interacted with Leu300 (TM7) and Ile304 (TM7), and Trp3 with Leu300 (TM7) and Ile304 (TM7) (Figure 9B). Similarly, in the simulation of DOR/camel hem-5 complex, Tyr1 exhibited hydrophobic interactions with Ile304 (TM7), and both Pro2 and Trp3 showed consistent hydrophobic interactions with Leu300 (TM7) and Ile304 (TM7) (Supplementary Figure 5B).

In the simulations, the positively charged Arg5 or Arg6 in the C terminus of hemorphin variants formed a stable salt bridge interaction with Asp210, a key residue in the DOR binding pocket. In the docked pose of the DOR/hem-7 complex, Gln5 formed hydrogen bonds with Ser206 and Asp210 in TM5, while in the DOR/hem-6 complex, Arg6 formed a hydrogen bond with Asp210 (TM5) (Figures 2A, B). Salt bridge interactions between Arg6 and Asp210 (TM5) were consistently observed in the MD simulations of both hem7 and hem-6 with DOR (Figures 6A, 7A). However, the simulations did not show a salt bridge interaction with Asp210 for hem-5 and hem-4, Instead, in DOR/hem-5, Gln5 showed hydrogen bond interactions with Arg192 in ECL2 and Arg291 in ECL3 (Supplementary Figure 4A). In the simulation runs of camel hem-7 with DOR, a stable salt bridge was observed between Arg6 and Asp210 (TM5), while in camel hem-6, Arg6 formed both a hydrogen bond and a salt bridge with Asp210 (TM5) (Figures 6B, 7B). In the case of camel hem-5, Arg5 (Q5R) formed a salt bridge with Asp210 (TM5) (Supplementary Figure 4A).

In addition to the salt bridge interaction with Asp210 (TM5), increased electrostatic interactions were noted with the arginine-rich C terminus of camel hemorphin variants. Specifically, Arg6 of camel hem-7 formed a salt bridge with Asp288 (ECL2), while Arg5 formed salt bridges with Asp108 (TM2) and Glu112 (ECL1) (Figure 6B). Arg5 of camel hem-6 formed salt bridges with Asp108 (TM2) and Asp193 (ECL2) while Arg5 of camel hem-5 formed a salt bridge with Asp193 (ECL2) (Figure 7B; Supplementary Figure 4B).

## 4 Discussion

The best binding conformation of hemorphins with the X-ray structure of human δ-opioid receptor were obtained with the peptide docking module of Schrodinger Maestro. Among the hemorphins docked, the longer forms of hemorphins, hem-7, camel hem-7, hem-6, and camel hem-6 showed strong interactions with DOR. However, based on the binding energy calculation, the camel variants, camel hem-7 and camel hem-6 showed high binding affinity towards DOR than the mammalian hemorphins. MD simulations highlighted the role and stability of specific interactions between hemorphins and DOR.

Tyr1 of all the hemorphin variants binds deeply within the hydrophobic binding pocket of DOR, forming multiple critical contacts with key amino acids responsible for the activation of the receptor. In the docked poses of all hemorphin variants with DOR, Tyr1 forms both hydrogen bond and salt-bridge interaction with Asp128, a crucial residue for receptor function. Specifically, the protonated amino group of Tyr1 engages in a salt-bridge interaction with the carboxyl group of Asp128 in the docked poses of all variants. Molecular dynamics simulations further confirm the stability of those interactions throughout the entire simulation time, suggesting a strong interaction between Tyr1 and Asp128. Most opioid receptor peptide agonists interact with DOR through a critical salt-bridge formation involving a basic, protonated nitrogen atom. This salt-bridge interaction is commonly observed between the protonated amine of opioid agonists and a conserved aspartate residue in the receptor, such as Asp128 (δ-opioid receptor), Asp138 (κ-opioid receptor), and Asp149 (μ-opioid receptor) (Huang et al., 2015; Che et al., 2018; Claff et al., 2019). This interaction is essential for receptor activation by these ligands and is a common feature in the binding pocket of opioid receptors (Roth et al., 2002; Claff et al., 2019). Claff et al. reported that opioid agonists with basic nitrogen that interacted with the conserved aspartate extend much deeper into the binding pocket than structurally similar antagonists. The positioning of the basic nitrogen deeper into the binding pocket is a hallmark of opioid agonist activity for ligands that contain a basic amine interacting with the conserved aspartate (Cavalli et al., 1999; Claff et al., 2019). Studies on opioid peptides have shown that acetylating or substituting the N-terminal amine abolished agonistic properties while maintaining low nanomolar affinity (Schiller et al., 2000; Ghosh et al., 2008). Additionally, Tyr1 consistently exhibited strong hydrophobic interactions with Tyr308, another key residue in DOR activation, in all the simulations. Asp128 is involved in a polar network with Thr101, Gln105, and Tyr308, linking TM helices 2, 3, and 5. This polar network surrounding the conserved Asp128 plays a crucial role in agonist-induced activation of DOR (Claff et al., 2019). Moreover, the presence of a peptide with an N-terminal tyrosine has been demonstrated to enhance the potency of peptides interacting with opioid receptors (Dumitrascuta et al., 2020; Sarkar et al., 2020; Ayoub and Vijayan, 2021; Lee, 2022). Peptides with this structural feature can aid in the specificity and affinity of the ligands for the δ-opioid receptor, enhancing their pharmacological effects. The tyrosine residue at the N-terminus of endogenous opioid peptides, such as enkephalins (Tyr-Gly-Gly-Phe-Leu or Met), is critical for their interaction with opioid receptors, as it forms key hydrogen bonds and hydrophobic contacts that facilitate receptor binding. This residue is essential for activating the signal transduction pathways of the receptor, leading to the analgesic effects of enkephalins (Weltrowska et al., 2010). Another peptide agonist, KGCHM07 that contains N-terminal tyrosine derivative 2,6-dimethyl-l-tyrosine (Dmt1), has been shown to enhance the binding affinity and activity of peptidic ligands at opioid receptors (Claff et al., 2019).

The extracellular loop 3 (ECL3) plays a critical role in peptide agonist selectivity and is notably nonconserved across different opioid receptors as illustrated in Figure 3D(Meng et al., 1996; Claff et al., 2019). During receptor activation, the ECL3 region undergoes significant conformational changes, which exposes the nonconserved Arg291 in ECL3 to the binding pocket. The involvement of the nonconserved Arg291 is particularly noteworthy, as it could act as a cationic counterpart to the carboxyl group of naturally occurring opioid peptides (Claff et al., 2019). This suggests a potential interaction between Arg291 and the “address” moiety of endogenous peptides, supporting the “message-address concept” proposed by Schwyzer in 1977 (Schwyzer, 1977). This concept hypothesizes that specific regions of a peptide, the “message,” are responsible for receptor activation, while other regions, the “address,” direct the peptide to the appropriate receptor. The role of Arg291 in this interaction enhances our understanding of the molecular mechanisms underlying opioid receptor selectivity and activation. Claff et al. reported that although, the peptide agonist, KGCHM07 activates both δ-opioid receptor and μ-opioid receptor (MOR), the DOR/KGCHM07 complex structure revealed the accessibility of Arg291 to the agonist binding pocket, as the μ-opioid receptor has a glutamic acid and the κ-opioid receptor has a histidine at this position (Figure 3D) (Claff et al., 2019). Extracellular loop 3 (ECL3), along with the extracellular ends of TM6 and TM7, formed a hydrophobic pocket that may be crucial for ligand binding or receptor activation. The hemorphin variants exhibited multiple contacts with nonconserved residues in ECL3 and the extracellular ends of TM6 and TM7. The interaction of Phe7 of hem-7 with Arg291 was found to be more stable compared to the interactions of other hemorphin variants with Arg291. The persistence of those interactions during the simulation suggests that the interaction of Phe7 and Arg291 are key contributors to the stability of the complex. The hemorphins showed more hydrophobic interactions with the nonconserved ECL3 and the nonconserved residues at the extracellular ends of TM6 and TM7 throughout the simulations. Trp3 maintained strong hydrophobic interactions, particularly with ECL3 and the nonconserved Trp284 (TM6) and Leu300 (TM7) in the simulations of all complexes. The widespread nature of these interactions suggests that Trp3 plays a significant role in stabilizing the interaction of hemorphins with the extracellular regions. These interactions highlight the importance of the aromatic and hydrophobic nature of Trp3 in stabilizing the hemorphin structure within the binding site. Interactions with the nonconserved residues might provide specific structural features unique to DOR, differentiating it from other receptors in the opioid receptor family.

Additionally, the hemorphins showed strong hydrophobic interactions with TM5 and TM7. The hydrophobic contacts with either helix 5 or helix 7, or both, prevent antagonists from deeply penetrating the binding pocket of DOR (Claff et al., 2019). MD simulations confirmed the stability of these hydrophobic interactions between the N-terminus (Tyr1-Pro2-Trp3) of hemorphins with TM5 and TM7.

A salt bridge consistently formed between the protonated amine group of the C terminal arginine and the carboxyl group of Asp210 (TM5), except in hem-5. Salt bridge interaction with Asp210 (TM5) serves as another crucial anchor point for the strong binding of agonist peptides to the δ-opioid receptor (Claff et al., 2019). The persistence of this salt bridge throughout the simulations highlights its importance in stabilizing the complex. The significance of this interaction is further underscored by experimental data showing that the mutation of Asp210Asn (D210N) leads to a 17-fold reduction in the binding affinity of the peptide agonist, KGCHM07, emphasizing the critical role of Asp210 in peptide binding (Claff et al., 2019). Hem-5 and hem-4 lack the positively charged arginine residue at the C terminus, preventing any electrostatic interactions with Asp210 (TM5). In the case of camel hem-5, Arg5 (Q5R) formed a salt bridge interaction with Asp210 (TM5). In contrast, hemorphin variants with positively charged residues at the C terminus, permitted electrostatic interactions with Asp210 (TM5), which enhances their binding stability.

Camel hemorphins exhibit distinct amino acid sequences compared to mammalian hemorphins, notably the substitution of glutamine with arginine (Q > R) in the C-terminal region. This substitution introduces a positively charged residue, facilitating additional electrostatic interactions. The observations of electrostatic interactions involving the arginine-rich C terminus of camel hemorphin variants highlight significant aspects of their binding dynamics and potential stability. Camel hemorphin-7 and camel hemorphin-6 exhibit strong binding affinity for the δ-opioid receptor by the formation of hydrogen bonds and electrostatic interactions with key residues such as Asp210. The presence of multiple salt bridges in the case of camel hemorphin variants likely enhances their binding affinity compared to mammalian hemorphins. These interactions not only indicate the importance of electrostatic forces in binding but also highlight the role of multiple charged residues in facilitating a strong interaction. The distinct patterns of salt bridge formation across the hemorphin variants may reflect differences in the binding mechanism or specificity with DOR. Overall, the increased electrostatic interactions observed with the arginine-rich C terminus of these camel hemorphin variants emphasize the critical role of electrostatic forces in stabilizing ligand-receptor interactions.

## 5 Conclusion

Peptide docking and molecular dynamics simulations we used to gain insights into the interaction of hemorphin variants with δ-opioid receptor. Camel hemorphin variants, camel hem-7, and camel hem-6, demonstrated strong binding towards DOR based on binding frees energy data. The N-terminal Tyr1 played a pivotal role in binding with DOR through critical interactions with key residues such as Asp128 and Tyr308. The deep binding of Tyr1 within the receptor, supported by stable hydrogen bonds and salt-bridges, underscores its importance in receptor activation. The interactions with nonconserved residues particularly with the nonconserved ECL3 and the extracellular ends of TM6 and TM7 further highlight the role of hemorphins in ligand selectivity and binding stability. Electrostatic interactions, especially those involving the C-terminal arginine-rich sequences in camel hemorphin variants, contributed to the enhanced binding affinity and stability of these peptides. The persistence of these interactions of hemorphin variants with DOR highlights its potential role in maintaining the stability of the hemorphin-DOR complexes, which can be assessed in future binding assays. These findings contribute to our understanding of the molecular basis of hemorphin interactions and provide insights which could be critical for designing therapeutic peptides with improved efficacy and specificity.

## Data Availability

The raw data supporting the conclusions of this article will be made available by the authors, without undue reservation.
