# Supplementary material for: Insights into the interaction between hemorphins and δ-opioid receptor from molecular modeling
Source: Front Mol Biosci. 2024 Dec 12;11:1514759. doi: 10.3389/fmolb.2024.1514759 (PMC11669586; doi:10.3389/fmolb.2024.1514759)
Supplement: Supplementary file 1 [file DataSheet1.pdf]

Insights into the interaction between hemorphins and δ-opioid receptor from molecular modeling

Priya Antony [1], Bincy Baby [1] and Ranjit Vijayan[1,2,3,*]

[1] Department of Biology, College of Science, United Arab Emirates University, Al Ain P.O. Box 15551, United Arab Emirates
[2] The Big Data Analytics Center, United Arab Emirates University, Al Ain P.O. Box 15551, United Arab Emirates
[3] Zayed Center for Health Sciences, United Arab Emirates University, Al Ain P.O. Box 15551, United Arab Emirates
* Correspondence: ranjit.v@uaeu.ac.ae (Ranjit Vijayan)

Supplementary Material

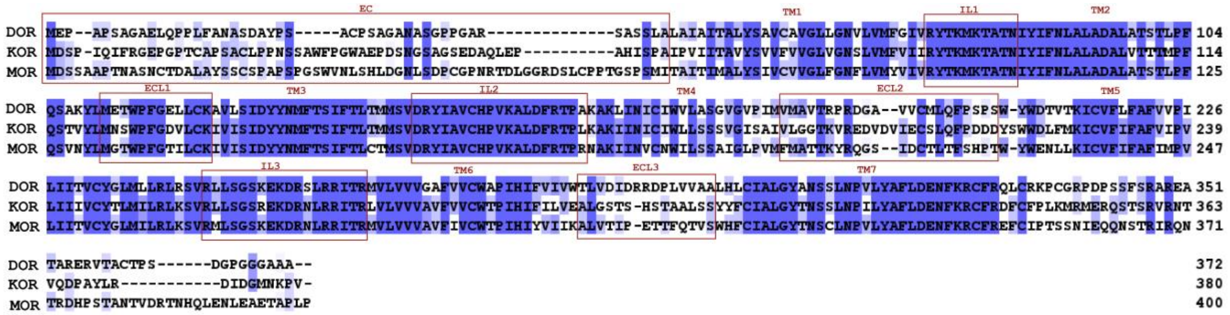

Supplementary Figure S1: Sequence alignment of δ-opioid receptor (DOR), κ-opioid receptor (KOR) and μ-opioid receptor (MOR).

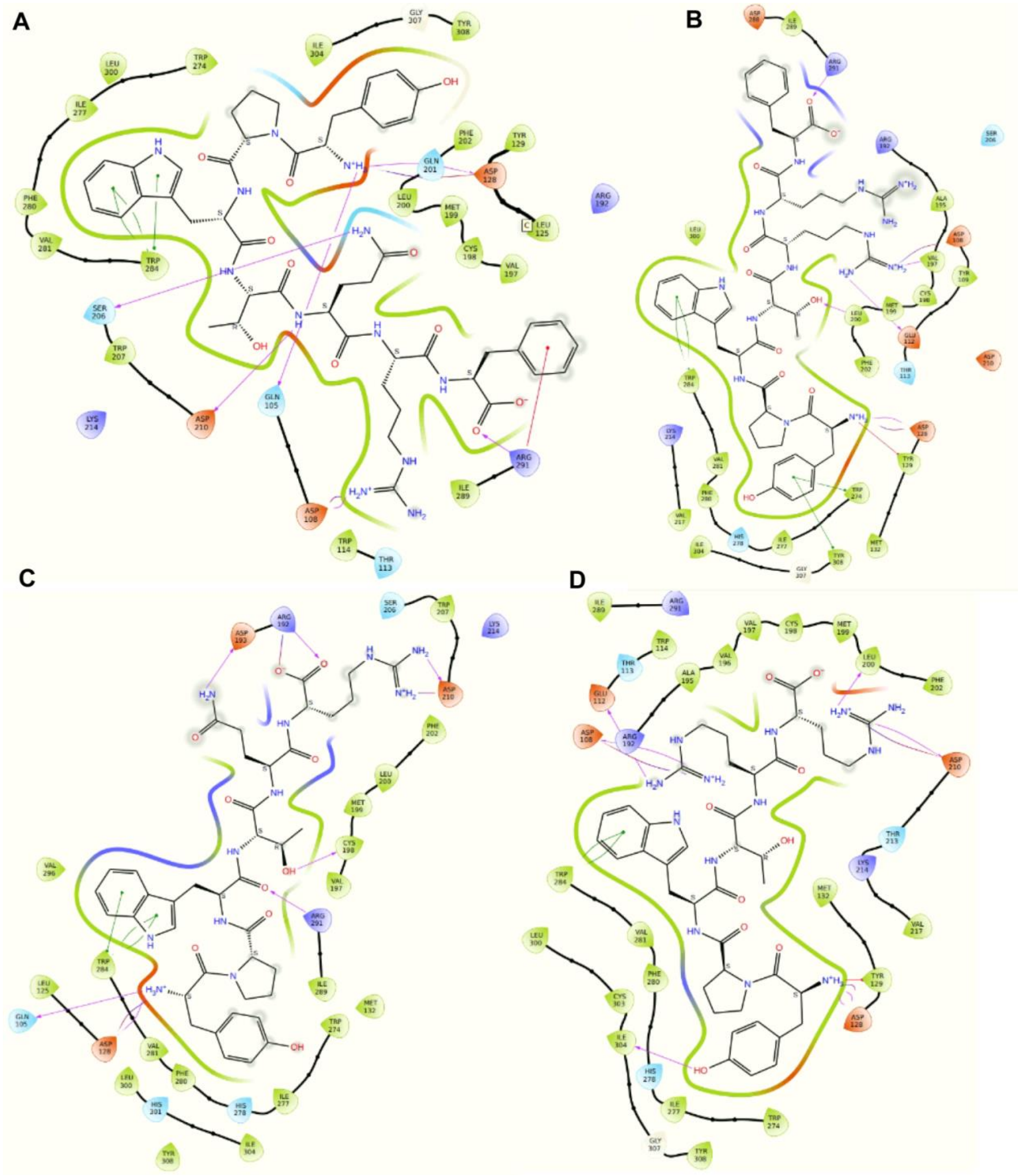

Supplementary Figure S2: The interaction of hemorphin variants with DOR in the molecular docking analysis. A) DOR/hem-7, B) DOR/camel hem-7, C) DOR/hem-6, and D) DOR/camel hem-6. Hydrogen bond (pink arrows), π-π interactions (green lines) π-cation (red lines), salt bridges (red-blue line), and the hydrophobic interacting residues are represented as green color residues.

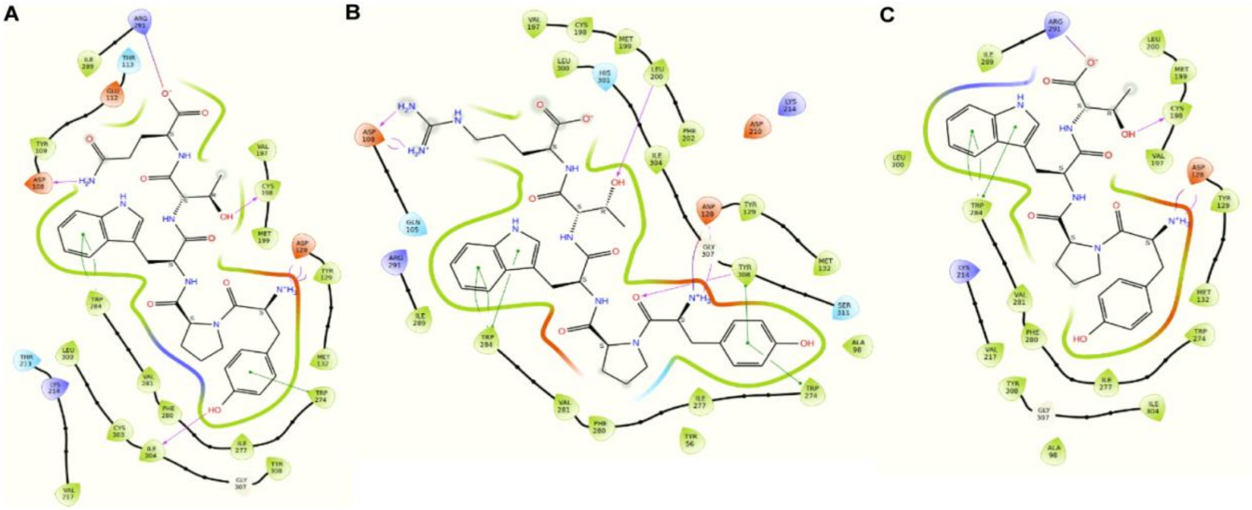

Supplementary Figure S3: The interaction of hemorphin variants with DOR in the molecular docking analysis. A) DOR/hem-5, B) DOR/camel hem-5, and C) DOR/hem-4. Hydrogen bond (pink arrows), π-π interactions (green lines) π-cation (red lines), salt bridges (red-blue line), and the hydrophobic interacting residues are represented as green color residues.

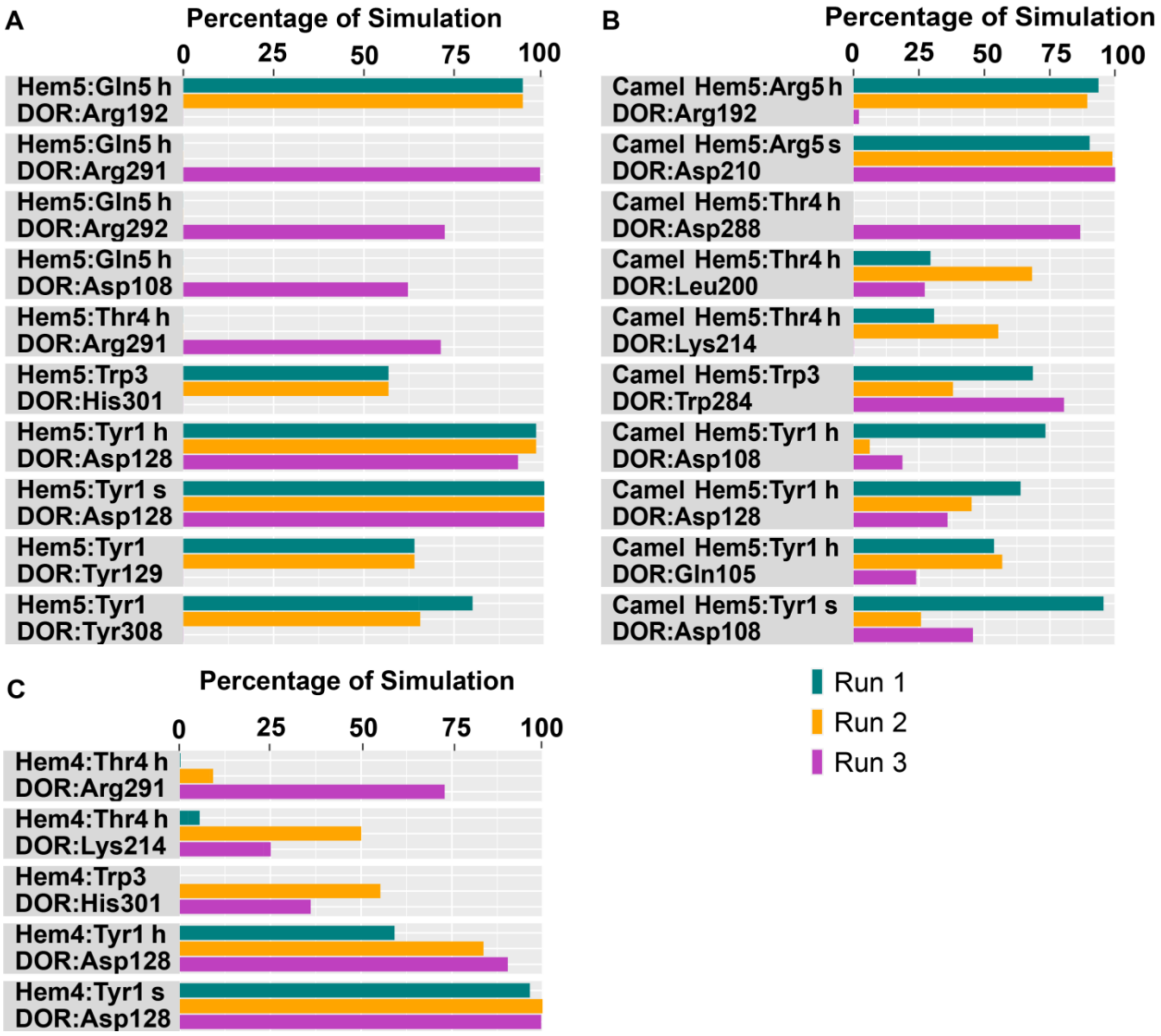

Supplementary Figure S4: Polar interactions between hemorphin variants and δ-opioid receptor. A) DOR/hem-5, B) DOR/camel hem-5, and C) DOR/hem-4.

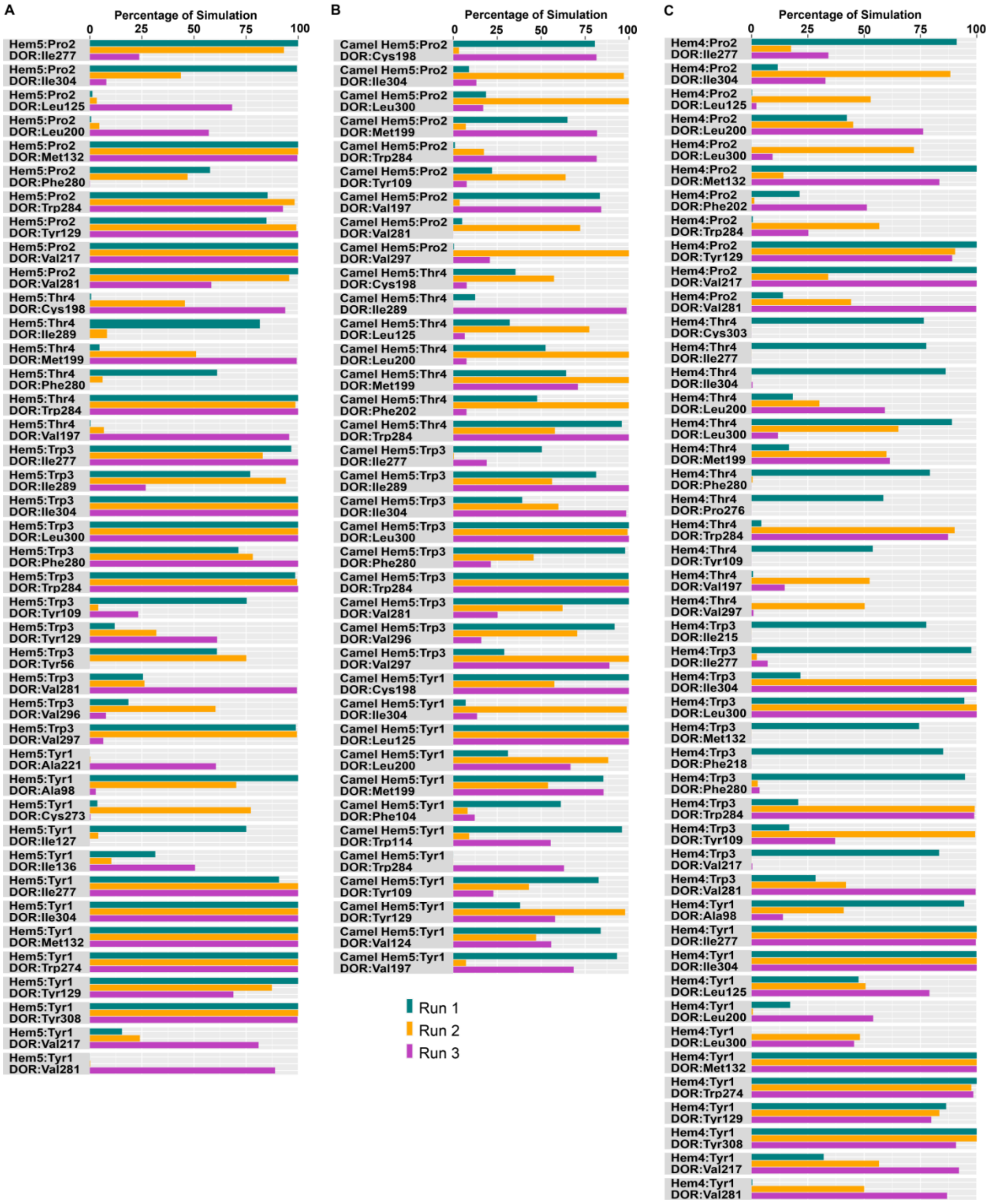

Supplementary Figure S5: Hydrophobic interactions between hemorphin variants and δ-opioid receptor. A) DOR/hem-5, B) DOR/camel hem-5, and C) DOR/hem-4.
